# Antioxidant, Antimicrobial and Antiproliferative Activities of Five Lichen Species

**DOI:** 10.3390/ijms12085428

**Published:** 2011-08-23

**Authors:** Tatjana Mitrović, Slaviša Stamenković, Vladimir Cvetković, Svetlana Tošić, Milan Stanković, Ivana Radojević, Olgica Stefanović, Ljiljana Čomić, Dragana Đačić, Milena Ćurčić, Snežana Marković

**Affiliations:** 1 Department of Biology and Ecology, Faculty of Science and Mathematics, University of Niš, 33, Višegradska, 18000 Niš, Serbia; E-Mails: sslavisa@pmf.ni.ac.rs (S.S.); biovlada@yahoo.com (V.C); tosicsvetlana59@yahoo.com (S.T.); 2 Department of Biology and Ecology, Faculty of Science, University of Kragujevac, 12, Radoja Domanovića, 34000 Kragujevac, Serbia; E-Mails: mstankovic@kg.ac.rs (M.S.); ivana@kg.ac.rs (I.R.); olgicas@gmail.com (O.S.); ljilja@kg.ac.rs (L.Č.); dragandjacic@kg.ac.rs (D.Đ.); milenagen@gmail.com (M.Ć.); smarkovic@kg.ac.rs (S.M.)

**Keywords:** lichens extract, total phenolic content, antioxidant activity, antimicrobial activity, antiproliferative activity

## Abstract

The antioxidative, antimicrobial and antiproliferative potentials of the methanol extracts of the lichen species *Parmelia sulcata, Flavoparmelia caperata, Evernia prunastri, Hypogymnia physodes* and *Cladonia foliacea* were evaluated. The total phenolic content of the tested extracts varied from 78.12 to 141.59 mg of gallic acid equivalent (GA)/g of extract and the total flavonoid content from 20.14 to 44.43 mg of rutin equivalent (Ru)/g of extract. The antioxidant capacities of the lichen extracts were determined by 2,2-diphenyl-1-picrylhydrazyl (DPPH) radicals scavenging. *Hypogymnia physodes* with the highest phenolic content showed the strongest DPPH radical scavenging effect. Further, the antimicrobial potential of the lichen extracts was determined by a microdilution method on 29 microorganisms, including 15 strains of bacteria, 10 species of filamentous fungi and 4 yeast species. A high antimicrobial activity of all the tested extracts was observed with more potent inhibitory effects on the growth of Gram (+) bacteria. The highest antimicrobial activity among lichens was demonstrated by *Hypogymnia physodes* and *Cladonia foliacea*. Finally, the antiproliferative activity of the lichen extracts was explored on the colon cancer adenocarcinoma cell line HCT-116 by MTT (3-[4,5-dimethylthiazol-2-yl]-2,5-diphenyltetrazolium bromide) viability assay and acridine orange/ethidium bromide staining. The methanol extracts of *Hypogymnia physodes* and *Cladonia foliacea* showed a better cytotoxic activity than the other extracts. All lichen species showed the ability to induce apoptosis of HCT-116 cells.

## 1. Introduction

Lichens are a unique life form of symbiosis between fungi (mycobionts) and algae and/or cyanobacteria (photobionts). They are considered to be the earliest colonizers of terrestrial habitats on the earth [1]. Nowadays, 25,000 different species of lichens inhabit over 10% of the terrestrial surface from arctic to tropical regions and from the plains to the highest mountains [2]. The specific, even extreme, conditions of their existence, slow growth and long duration (maximum lifetime spans to several thousand years) are consistent with their abundance in protective metabolites against different physical and biological influences [3].

Generally, lichens metabolites can be divided into two groups: primary and secondary. Primary metabolites are proteins, lipids, carbohydrates and other organic compounds involved in lichen’s metabolism and structure. Secondary metabolites, known as lichens substances, are mostly small, but complex molecules. Structures for more than 1050 different lichen substances have been reported to date [4]. They are produced by the fungus or the alga *per se*, while others are exclusively produced by synergistic action of both partners in lichens. Secondary metabolites are usually insoluble in water and can be extracted into organic solvents. Their amount ranges from 0.1 to 10% of the dry weight of tallus and sometimes reaches 30% [2]. Secondary metabolites exert a remarkable variety of biological effects: antiviral, antibacterial, antifungal, antiprotozoal, antiherbivore, antimutagenic, antioxidant, antitumor, antiulcerogenic, antinociceptive, antipyretic and anti-inflammatory activities. These effects were exploited in traditional medicine for treatment of various conditions (external wounds, burns, gastritis, cold, asthma, tuberculosis, *etc*.) in humans and animals since Egyptian times.

Nowadays, the imbalance between intracellular antioxidants and intracellular reactive oxygen species (ROS) or the so-called state of oxidative stress is a known contributing factor to over a hundred diseases. Antioxidants prevent oxidative damage of biomolecules and cells and ROS-induced diseases by reacting with free radicals, scavenging free radicals and chelating free catalytic metals [5]. The prevention with synthetic antioxidants (butylated hydroxyanisole (BHA), butylated hydroxytoluene (BHT), tert-butylhydroquinone (TBHQ) and propyl gallate (PG)) exerts a toxic and carcinogenic effect [6]. A strong antioxidant power of some lichen species was demonstrated in several studies [7–14].

The growing population of drug-resistant microorganisms and the problem of treating the infections induced have motivated the search for alternative antimicrobial drugs in lichens. The antibacterial activity against Gram (+) and Gram (–) bacteria, as well as the antifungal activity is shown for many lichen species [2,15–21].

Furthermore, the antitumor potential of lichen flora is investigated. Perry *et al*. screened a collection of 69 lichen species for their antiproliferative activity [22]. A high proportion of the lichen extracts manifested a cytotoxic activity against BS-C-1 (African green monkey kidney) cells and/or P388 (murine leukemia) cells. Ten lichen substances were reported as cytotoxic [23]. The most famous among them are: usnic acid, protolichesterinic- and lobaric acids [24–26].

The aim of this study is the evaluation of the antioxidant, antimicrobial and antiproliferative capacities of the most abundant lichen species in the southeast of Serbia (*Parmelia sulcata, Flavoparmelia caperata, Evernia prunastri, Hypogymnia physodes* and *Cladonia foliacea*).

## 2. Results and Discussion

### 2.1. Total Phenolic Content, Total Flavonoid Content and Antioxidant Activity

The antioxidant potential of methanol extract of *Parmelia sulcata, Flavoparmelia caperata, Evernia prunastri, Hypogymnia physodes* and *Cladonia foliacea* was estimated by determining their total phenolic and flavonoid contents and their ability for free radical scavenging. The results are shown in Table 1.

The total phenolic content of studied lichens extracts were determined by Folin-Ciocalteu method [33]. The amount of phenolic compounds varied from 78.12 to 141.59 mg GA/g of extract. The highest phenolic content was found in *Hypogymnia physodes* and the lowest in *Cladonia foliacea*. The amount of phenolic compounds in *Evernia prunastri* was approximately the same as the amount in *Cladonia foliacea. Parmelia sulcata* and *Flavoparmelia caperata* showed close values of phenolic content.

The total flavonoid content was evaluated using aluminum chloride [34]. The amount of flavonoid compounds ranged from 20.14 to 44.43 mg Ru/g of lichen extract. The highest flavonoid content was identified in *Parmelia sulcata* and the lowest in *Hypogymnia physodes. Evernia prunastri* and *Cladonia foliacea* had approximately the same values of the total flavonoid content.

DPPH radical scavenging capacities of lichen were measured by the modified method of Tekao *et al.* [35,36]. The observed values of IC_50_, *i.e*., the concentration of extract decreasing the initial DPPH concentration to 50%, varied from 45.57 to >1000.00 μg/mL. The DPPH radical scavenging capacity of *Hypogymnia physodes* was significantly higher than the capacity of the other four samples (45.57 μg/mL). *Evernia prunastri* and *Cladonia foliacea* showed the lowest scavenging capacity. *Parmelia sulcata* and *Flavoparmelia caperata* showed a similar ability for scavenging DPPH radicals.

The antioxidant activity of lichen species *Evernia prunastri* and *Cladonia foliacea* has not been previously investigated. The comparison of the chemical content of the tested extracts and their free radical scavenging ability revealed a strong correlation which was in accordance with the previous results from Rankovic, Kosanic and colleagues [12,13]. An oposite finding of Odabasoglu *et al.* with methanol extracts of *Lobaria pulmonaria* and *Usnea longissima,* were explained by the participation of other, non-polar components, insoluble in methanol in this activity [37].

A list of compounds previously detected in methanol extracts of *Parmelia sulcata, Flavoparmelia caperata, Evernia prunastri, Hypogymnia physodes* and *Cladonia foliacea* is given in Table 1. Lichen phenolic substances–depsides, depsidones and dibenzofurans, are well known for their antioxidant activities [12,35,36]. Also, one should have in mind that the concentration of antioxidants fluctuates with environmental conditions. Extreme environmental conditions (high temperature, high light, desiccation, rehydratation, air pollution) reduce synthesis of antioxidants in lichens and therefore decrease its antioxidant activity [39–41].

### 2.2. Antimicrobial Activity

The results of *in vitro* testing of the antibacterial and antifungal activities of the methanol extracts of lichens *Parmelia sulcata, Flavoparmelia caperata, Evernia prunastri, Hypogymnia physodes* and *Cladonia foliacea* are shown in Tables 2–5.

The antimicrobial activity of lichen extracts was evaluated by microdilution method with resazurin [42]. The minimum inhibitory concentrations (MIC) and the minimum microbicidal concentrations (MMC) of extracts were determined on the collection of 29 microorganisms including 15 strains of bacteria, 10 species of filamentous fungi and 4 species of yeasts. MIC and MMC values ranged from 9.8 × 10^−3^ mg/mL to 40.00 mg/mL (Tables 2–5). The tested extracts showed different levels of antimicrobial activity depending on the group of microorganisms (Gram(+), Gram(–), bacteria, filamentous fungi, yeasts) and the species of lichens. In general, lichen methanol extracts demonstrated a high antimicrobial activity. Their inhibitory effect was the most potent on Gram (+) bacteria and the weakest on yeasts. Three lichen species *Evernia prunastri*, *Hypogymnia physodes* and *Cladonia foliacea* manifested the strongest antimicrobial activity (p < 0.05).

The analysis of antibacterial activity indicated *Hypogymnia physodes* and *Cladonia foliacea* as the most potent extracts and *Parmelia sulcata* as the weakest one (Table 2 and Table 3). *Hypogymnia physodes* demonstrated the strongest activity on Gram (+) bacteria *Sarcina lutea* (MIC and MMC values <9.8 × 10^−3^ mg/mL) and *Staphyloccocus aureus* (MIC and MMC values 3.91 × 10^−2^ and 7.81 × 10^−2^ mg/mL) (Table 3). Also, a high activity was shown on Gram (–) bacteria *Pseudomonas aeruginosa*, *Pseudomonas aeruginosa* ATCC 27853 and *Proteus mirabilis* with the same MIC values (6.25 × 10^−1^ mg/mL) and on *Salmonella typhymirium* with higher MIC value (1.25 mg/mL) (Table 3). The extract of *Cladonia foliacea* was the most effective against Gram (+) bacteria *Bacillus subtilis*, *Bacillus subtilis* ATCC 6633 and *Bacillus cereus* with the same MIC and MMC value <9.8 × 10^−3^ mg/mL (Table 3). The most resistant Gram (+) bacteria was *Enterococcus faecalis* and the most resistant Gram (–) bacteria was *Escherichia coli.*

In general, the antifungal activity of the tested lichen extracts was less prominent. The best results were obtained with *Evernia prunastri* and *Hypogymnia physodes* extracts. *Evernia prunastri* showed the best effect on yeasts (Table 4). Both, *Evernia prunastri* and *Hypogymnia physodes,* were active against *Rhodotorula* sp. with the same MIC and MMC values (1.25 and 2.5 mg/mL, respectively) (Table 4 and Table 5). Aditionally, *Evernia prunastri* showed good results with filamentous fungi, especially *Aspergillus niger* ATCC 16404 with both MIC and MMC values of 1.56 × 10^−1^ mg/mL (Table 4). Concerning *Hypogymnia physodes,* it demonstrated the highest activity on filamentous fungi. The best effect was observed on *Aspergillus niger* ATCC 16404 (MIC and MMC values <9.8 × 10^−3^ mg/mL) (Table 5). Also, *Hypogymnia physodes* was the most potent against all representatives of *Penicillium* species (Table 5).

Mostly, lichen extracts demonstrated a similar activity against standard and clinical strains of the same microorganism. Few exceptions were registered. Four out of five lichen species tested (*Flavoparmelia caperata*, *Evernia prunastri*, *Hypogymnia physodes* and *Cladonia foliacea*) acted differently on the standard and clinical bacterial strain of *Enterococcus faecalis* (Table 2 and Table 3). The extract of *Parmelia sulcata* showed a different effect on the standard and clinical bacterial strain of *Staphylococcus aureus* (Table 2). The extract of *Hypogymnia physodes* demonstrated a variability in affecting the standard and clinical strain of filamentous fungi *Aspergillus niger* (Table 5).

The antimicrobial activity of the tested lichen species was examined in several studies in various ways and with different results. Ranković and colleagues observed the dependence of the level of the antimicrobial activity of the same lichen species on the solvent used in extraction [17]. They emphasized the strongest antimicrobial activity of the methanol extracts compared to the extracts in other solvents. This is consistent with the observation of Bezivin *et al.* that polar lichen compounds were mostly found in the methanol extract [23]. Besides this, the differences in previous studies could reflect: different quantity of the same active component in lichen extracts, different components involved in antimicrobial actions, different locations of lichen sampling, and different sensitivity of tested microorganisms or different methods of testing. Cansaran-Duman *et al.* underlined the dependence between a quantity of usnic acid possessed and the antimicrobial effect exhibited by various *Hypogymnia* species [43]. The higher the content of usnic acid in the species tested, the stronger the antimicrobial activity observed. Candan *et al.* connected the antimicrobial activities of *Parmelia sulcata* with salazinic acid constituents [44].

Our study of the antimicrobial properties of the methanol extracts of lichens *Parmelia sulcata, Flavoparmelia caperata, Evernia prunastri, Hypogymnia physodes* and *Cladonia foliacea,* showed a different degree of antimicrobial activity depending on the tested group of microorganisms and the tested species. This is the first study of the antimicrobial activity of *Evernia prunastri* and *Cladonia foliacea* originating from our territory. Generally, the tested extracts demonstrated a good antimicrobial activity. Our study confirmed the highest antimicrobial activity of *Hypogymnia physodes* earlier noticed by Ranković and colleagues [17–21]. Also, the tested extracts showed more potent inhibitory effects on Gram (+) bacteria than on other microorganisms, due to their specificity of the cell wall structure.

### 2.3. Antiproliferative Activity

**Figure 1.**
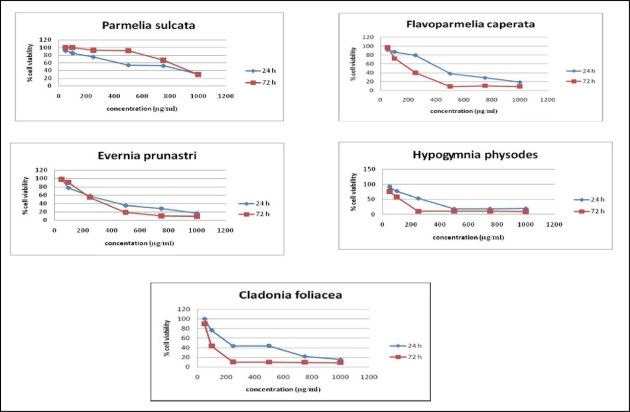
The dose-response effect of lichen extracts on HCT-116 cells growth. The cells were treated with methanol extract in concentration range from 50–1000 μg/mL. The antiproliferative effects were measured by MTT assay after 24 and 72 h exposure. Results were expressed as the means ± SE from three independent experiments.

The antiproliferative activity of methanol extracts of *Parmelia sulcata, Flavoparmelia caperata, Evernia prunastri, Hypogymnia physodes* and *Cladonia foliacea* was evaluated by the MTT viability assay and the acridine orange/ethidium bromide (AO/EB) double staining [45,46]. The colon cancer adenocarcinoma cell line HCT-116 was exposed to the various concentration of extract (50–1000 μg/mL) for a period of 24 h and 72 h. After the treatment cell viability was measured by the MTT reduction assay. The results of the assay are represented in Figure 1.

The extract of *Parmelia sulcata* did not induce a significant inhibition of cell growth in a dose- and time-dependent manner (Figure 1). The maximal inhibition was observed for the concentration of 1000 μg/mL after 24 and 72 h exposure. Extracts of *Flavoparmelia caperata, Hypogymnia physode*s and *Cladonia foliacea* demonstrated a significant inhibition of cell growth in a dose- and time-dependent manner (Figure 1). Hence, the higher the concentration of the extract applied, the higher cell sensitivity observed. The longer the time of the exposure, the higher cell sensitivity induced. Finally, extracts of *Evernia prunastri* manifested cell viability reduction in a dose-dependent manner. For a longer time of treatment (72 h), a higher cell sensitivity is observed, except with lower concentrations of extract (Figure 1). The comparison of the percentage of viable cells after 24 and after 72 h, revealed a time-dependent reduction of cell viability for higher concentrations.

The antiproliferative effect of each extract was expressed by IC_50_(inhibitory dose which inhibits 50% of cell growth) (Table 6). According to the American National Cancer Institute (NCI), a crude extract may be considered as active for an IC_50_< 30 μg/mL [47]. Based on this criterion, active substances in methanol extracts from *Parmelia sulcata, Flavoparmelia caperata, Evernia prunastri, Hypogymnia physodes* and *Cladonia foliacea* could not be described as cytotoxic.

The ability of the lichen extracts to induce apoptosis was screened by the acridine orange/ethidium bromide staining. According to the fluorescence emission and the morphological aspect of chromatin condensation in stained nuclei, four types of cells could be distinguished [46]. Viable cells (VC) possessed uniform bright green nuclei with organized structure and orange cytoplasm. Early apoptotic cells (EA), with intact membranes and initial DNA cleavage, were characterized by green nuclei with perinuclear chromatin condensation visible as bright green patches or fragments. Late apoptotic cells (LA) were recognized by orange to red nuclei with condensed or fragmented chromatin. Necrotic cells (N) exhibited uniformly orange to red nuclei with organized structure.

**Figure 2.**
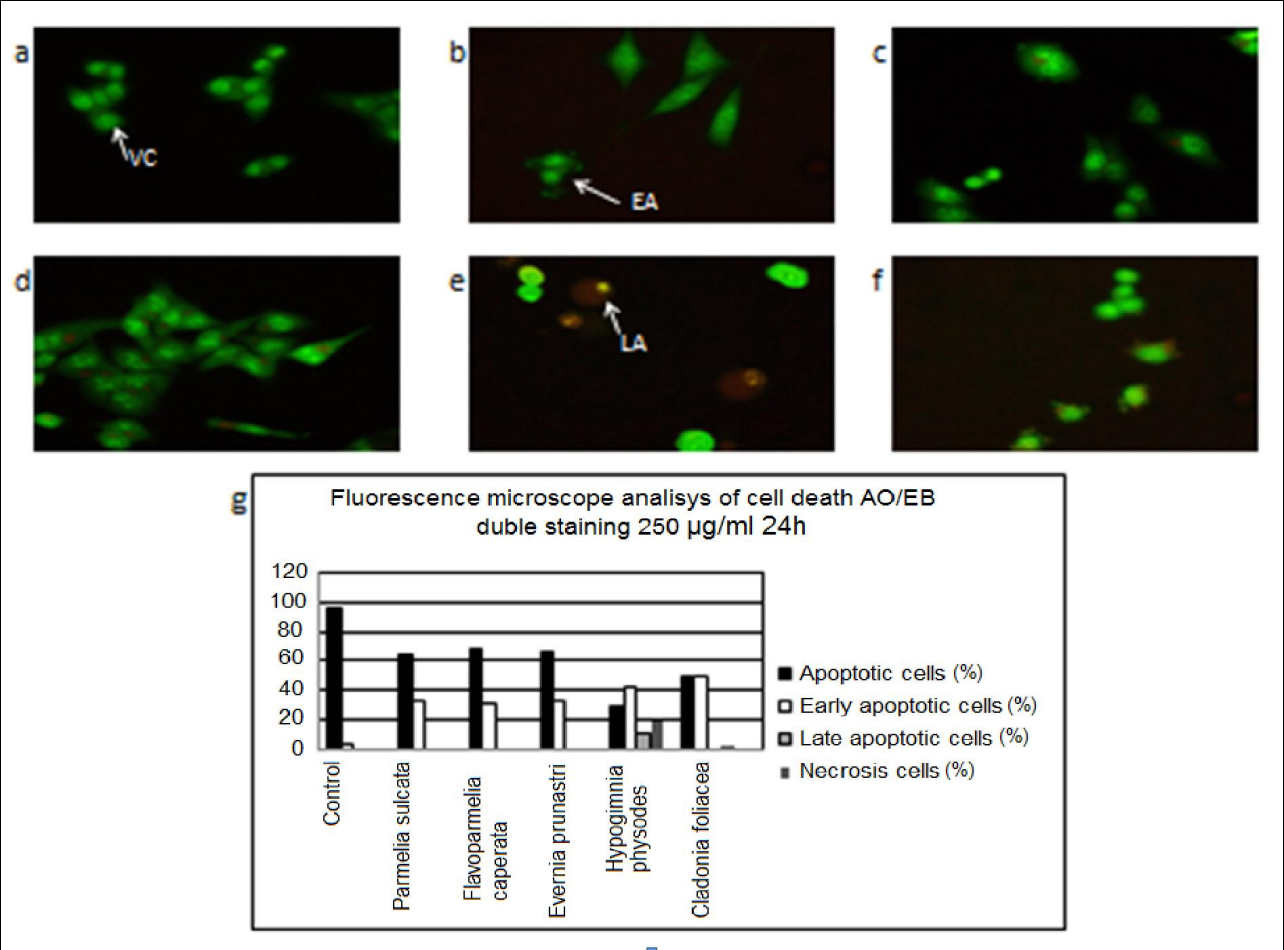
The effect of the lichen extracts on the apoptosis of HCT-116 cells after 24 h exposure monitored by the acridine orange/ethidium bromide staining: (**a**) Untreated, control cells; (**b**) Cells treated with *Parmelia sulcata* extract; (**c**) Cells treated with *Flavoparmelia caperata* extract; (**d**) Cells treated with *Evernia prunastri* extract; (**e**) Cells treated with *Hypogymnia physodes* extract; (**f**) Cells treated with *Cladonia foliacea* extract. Magnification on fluorescent microscope was 400×; (**g**) Grafic representation of obtained data. VC – viable cell, EA – early apoptotic cell, LA – late apoptotic cell.

**Figure 3.**
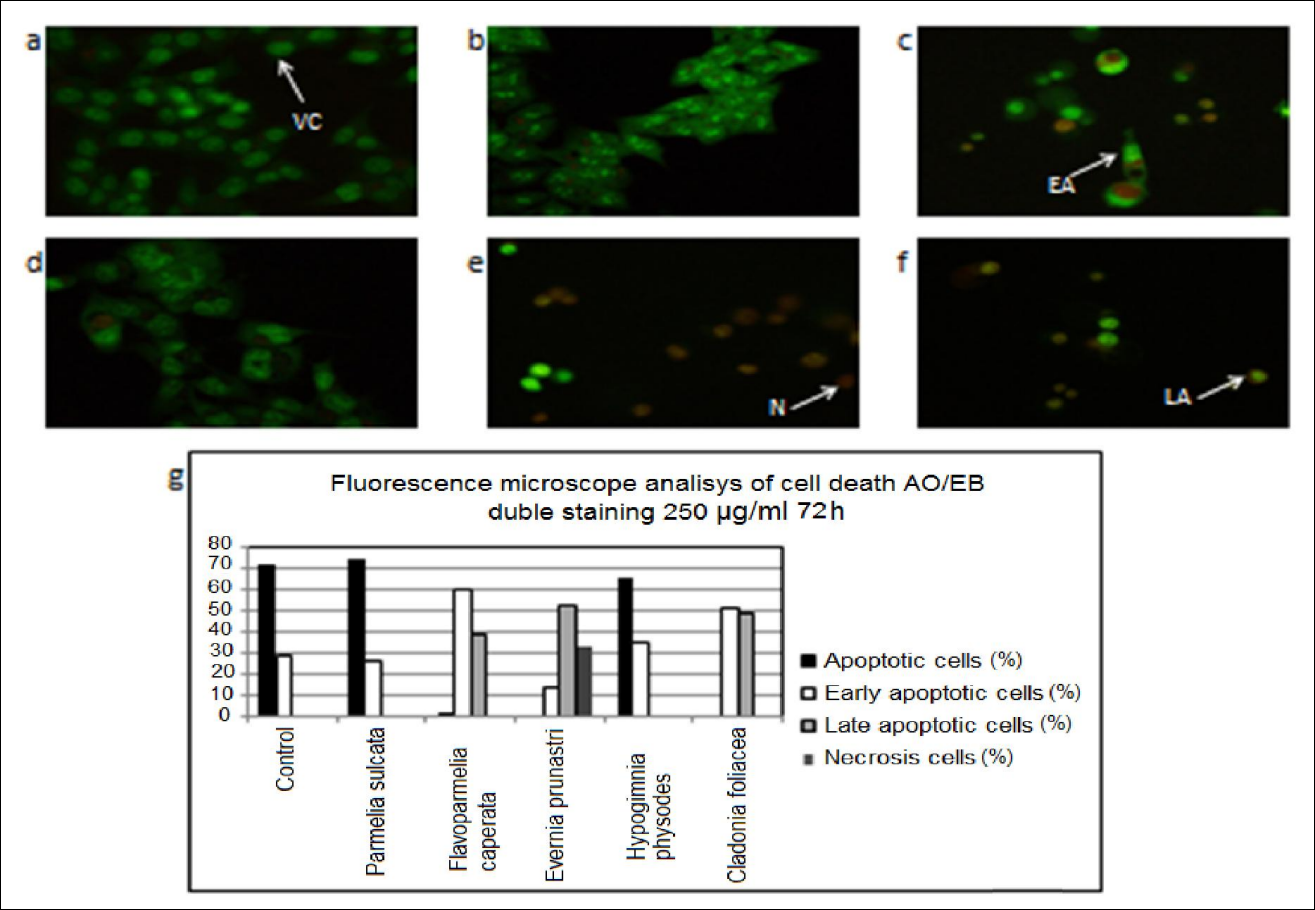
The effect of the lichen extracts on apoptosis of HCT-116 cells after 72 h exposure monitored by the acridine orange/ethidium bromide staining: (**a**) Untreated, control cells; (**b**) Cells treated with *Parmelia sulcata* extract; (**c**) Cells treated with *Flavoparmelia caperata* extract; (**d**) Cells treated with *Evernia prunastri* extract; (**e**) Cells treated with *Hypogymnia physodes* extract; (**f**) Cells treated with *Cladonia foliacea* extract. Magnification on fluorescent microscope was 400×; (**g**) Grafic representation of obtained data. VC – viable cell, EA – early apoptotic cell, LA – late apoptotic cell, N – necrotic cell.

The results obtained with the acridine orange/ethidium bromide (AO/EB) staining of HCT-116 cells exposed 24 h to 250 μg/mL of various lichen extracts are shown in Figure 2 and Table 7 while the results for 72 h treatment are shown in Figure 3 and Table 8. The untreated, control HCT-116 cells were characterized by bright green nucleus with uniform intensity and the absence of ethidium bromide uptake, while apoptotic cells appeared orange (Figure 2a). HCT-116 cells treated with lichen extracts from all five species showed obvious nuclear condensation after 24 h of treatment (Figure 2b–f). Fluorescence microscopic images clearly revealed nuclear disintegration of the treated cells compared to the untreated control cells. Compared with the spontaneus apoptosis observed in the control cells (early apoptotic 3.20%, 0% late apoptotic and 0% necrotic cells), HCT-116 cells treated with extracts of all lichen species showed increased percentages of early apoptotic cells for 24 h treatment. The extract of *Hypogymnia physodes* with the highest antiproliferative potential and IC_50_of 253.72 μg/mL (Table 7) showed increased percentages of early apoptotic (42.22%), late apoptotic (11.11%) and necrotic cells (17.78%) after 24 h (Figure 2e). The extract of *Cladonia foliacea* showed maximal induction of early apoptotic phase (49.66%) (Figure 2f).

A longer exposure (72 h) of HCT-116 cells to the lichen extract enhanced apoptosis (Figure 3 and Table 8). Compared to the spontaneous apoptosis observed in control cells (early apoptotic cells 28.80%, 0% late apoptotic and 0% necrotic cells), a progress toward late apoptosis and an obvious nuclear condensation were noticed in the treated cells. The extract of *Hypogymnia physodes* showed the most prominent effect by increasing late apoptosis (53.15%) and necrosis (32.62%) (IC_50_= 102.40 μg/mL) (Figure 3e). The extract of *Cladonia foliacea* exerted progression toward late apoptosis (48.40%) (Figure 3f).

Our study is the first attempt to evaluate the antiproliferative activity of lichen species on our territory. Bezivin and colleagues investigated cytotoxic activity of eight French lichen species, including *Parmelia caperata* and *Evernia prunastri* [23]. They performed extraction with solvents of increasing polarity (n-hexane, diethyl ether and methanol). Although the percentage of apolar, mid-polar and polar compounds was different between species, some similarities inside a genus were observed. The highest quantities of compounds were extracted with methanol regardless of the lichen species. n-hexane fraction of *Parmelia caperata* was the most active on DU145 (human brain metastasis of prostate carcinoma) cells whereas methanol fraction was selectively cytotoxic on DU145, 3LL (murine Lewis lung carcinoma) and U251 (human glioblastoma) cells. n-hexane extract of *Evernia prunastri* demonstrated cititoxicity on DU145 cells and its methanol extract on 3LL cells. Bezivin *et al.* considered the involvement of usnic acid, as a major compound of n-hexane fraction of the mentioned lichen species, in cytotoxic activity on cancer cell lines.

An extract of *Hypogymnia physodes* and *Cladonia foliacea* with their prominent apoptotic potential in this study could be useful as a desirable strategy for cancer control, similar to many commercially available chemotherapeutic agents and folk medicinal plants. Having in mind *Hypogymnia physodes* abundance in phenolic compounds and its antioxidative power, it could be considered as a cotreatment with some stronger cytotoxic agents, chemotherapy agents (for example cisplatin). Previous reports demonstrated that many side effects of the commonly used chemotherapy agents are a consequence of the induction of oxidative stress, which could be palliated by antioxidant food and plants uptake [48]. However, there is a need to fully substantiate the findings through future comprehensive studies of *Hypogymnia physodes* and *Cladonia foliacea* extracts. To further determine the activity and mechanism of their action, we should isolate and identify the active principle(s).

## 3. Experimental Section

### 3.1. Chemicals

Acetone, methanol, ethyl acetate and sodium hydrogen carbonate were purchased from “Zorka Pharma” Šabac, Serbia. Gallic acid, rutin hydrate, chlorogenic acid and 2,2-diphenyl-1-picrylhydrazyl were obtained from Sigma Chemicals Co., St Louis, MO, USA. Folin-Ciocalteu phenol reagent and aluminium chloride hexahydrate were purchased from Fluka Chemie AG, Buchs, Switzerland. Nutrient liquid medium for microorganisms, a Mueller–Hinton broth was obtained from Liofilchem, Italy, while a Sabouraud dextrose broth was obtained from Torlak, Belgrade. An antibiotic doxycycline was purchased from Galenika A.D., Belgrade, Serbia and antimycotic fluconazole from Pfizer Inc., USA. Dulbecco’s Modified Eagle Medium, fetal bovine serum, penicillin and streptomycin were obtained from Gibco, Invitrogen, New York, USA. 3-[4,5-dimethylthiazol-2-yl]-2,5-diphenyltetrazolium bromide and dimethyl sulfoxide were purchased Sigma, St. Louis, USA. All other solvents and chemicals were of analytical grade.

### 3.2. Lichen Material

Corticolous lichens species: *Hypogymnia physodes* (L.) Nyl., (syn: *Parmelia duplicata* var. *douglasicola* Gyelnik, *Parmelia physodes* (L.) Ach., *Parmelia oregana* Gyelnik; common names: Monk's-hood lichen, Hooded tube lichen, Puffed lichen), *Evernia prunastri* (L.) Ach. **(**common name: oakmoss), *Flavoparmelia caperata* (L.) Hale (syn: *Parmelia caperata* (L.) Ach.; common name: greenshield lichen), *Parmelia sulcata* Taylor (common name: shield lichen), growing on the *Prunus domestica* and *Salix sp.,* were collected in the southeast of Serbia in April 2009. The collection site is Bojanine vode near Niš at 860 m. Terricolous lichen species *Cladonia foliacea* (syn: *Cladonia alcicornis* (Leightf.) Fr.) was collected at Jelašnička klisura at 330 m. To determinate lichens we used several standard keys [49–51]. Lichen samples were deposited in the lichenological herbarium of the Department of Biology and Ecology, Faculty of Sciences and Mathematics, University of Niš.

### 3.3. Preparation of Lichen Extracts

Air-dried lichen thalli were ground (10 g of material of each species separately). Extractions were performed with 250 mL of methanol at room temperature for a period of 24 h. The extracts were filtered using Whatman No.1 filter paper and then concentrated in rotary vacuum evaporator at 40 °C.

### 3.4. Total Phenolic Content, Toral Flavonoid Content and Antioxidant Activity

#### 3.4.1. Determination of Total Phenolic Content

The total phenolic content of the lichen extracts was determined spectrophotometrically by Folin-Ciocalteu method [33]. Briefly, 0.5 mL of methanol extract solution (1 mg/mL) and 2.5 mL of 1:10 Folin-Ciocalteau reagent (Fluka Chemie AG, Buchs, Switzerland) were mixed and than 2 mL of sodium carbonate (75 g/L) were added. After 15 min of incubation at 45 °C, the absorbance at 765 nm was measured (ISKRA, MA9523-SPEKOL 211). The total phenolic concentration was calculated from gallic acid (GA) (Sigma Chemicals Co., St Louis, MO, USA) calibration curve. Data were expressed as gallic acid equivalents (GA)/g of extract averaged from 3 measurements.

#### 3.4.2. Determination of Total Flavonoid Content

The total flavonoid content was evaluated using aluminum chloride [34]. The sample for determination was prepared by mixing a 1 mL of methanol extract solution (1 mg/mL) and 1 mL of aluminum chloride (20 g/L). After 1 h of incubation at room temperature, the absorbance at 415 nm was measured (ISKRA, MA9523-SPEKOL 211). The total flavonoid concentration in lichen extract was calculated from rutin (Ru) (Sigma Chemicals Co., St Louis, MO, USA) calibration curve and expressed as rutin equivalents (Ru)/g of dry extract. Measurements were done in triplicates.

#### 3.4.3. Determination of Free Radical Scavenging Activity

The antioxidant activity of lichen extract was evaluated according to scavenging activity of stable radical 2,2-diphenyl-1-picrylhydrazyl (DPPH) (Sigma Chemicals Co., MO, St Louis, USA). DPPH assay was performed by a modified method of [35,36]. Serial dilutions of the extract were made from 1000 μg/mL to 0.97 μg/mL. 1 mL of each dilution was mixed with 80 μg/mL DPPH. After 30 min of incubation in darkness at room temperature, the absorbance was measured at 517 nm (ISKRA, MA9523-SPEKOL 211). The control sample contained all the reagents except the extract. The percentage of inhibition was calculated using the following equation:

(1) $$\%\;\mathrm{inhibition}=\left(\frac{A\;\text{control}-A\;\text{sample}}{A\;\text{control}}\right)\times 100$$

where A control was the absorbance of the control sample and A sample is the absorbance of extract. IC_50_values (concentration of the extract in the reaction mixture which decrease the initial DPPH concentration to 50%) were estimated from % inhibition *versus* the concentration sigmoidal curve using non-linear regression analysis. The data were presented as mean values ± standard deviation (n = 3).Chlorogenic acid was used as standard (IC_50_value 11.65 ± 0.52).

### 3.5. In Vitro Antimicrobial Assays

#### 3.5.1. Test Substances

Lichen extracts were dissolved in DMSO and then diluted into nutrient liquid medium to achieve a concentration of 5% DMSO. An antibiotic doxycycline (Galenika A.D., Belgrade, Serbia) was dissolved in nutrient liquid medium, a Mueller–Hinton broth (Torlak, Beograde, Serbia), while an antimycotic fluconazole (Pfizer Inc., USA) was dissolved in Sabouraud dextrose broth (Torlak, Belgrade, Serbia).

#### 3.5.2. Test Microorganisms

The antimicrobial activity of methanol extracts of five lichens (*Parmelia sulcata, Flavoparmelia caperata, Evernia prunastri, Hypogymnia physodes* and *Cladonia foliacea*) was tested against 29 microorganisms including the 15 strains of bacteria (standard strains: *Escherichia coli* ATCC 25922, *Staphylococcus aureus* ATCC 25923, *Enterococcus faecalis* ATCC 29212, *Pseudomonas aeruginosa* ATCC 27853, *Bacillus subtilis* ATCC 6633, and clinical strains: *Escherichia coli, Staphylococcus aureus, Enterococcus faecalis*, *Pseudomonas aeruginosa*, *Proteus mirabilis, Sarcina lutea, Salmonella enterica, Salmonella typhymirium*, *Bacillus subtilis and Bacillus cereus*); 10 species of filamentous fungi: *Aspergillus niger* ATCC 16404, *Aspergillus fumigatus* PMFKG-F23, *Aspergillus flavus* PMFKG-F24, *Aspergillus restrictus* PMFKG-F25, *Aspergillus niger* PMFKG-F26, *Penicillium italicum* PMFKG-F29, *Penicillium digitatum* PMFKG-F30, *Penicillium chrysogenum* PMFKG-F31, *Trichothecium roseum* PMFKG-F32, *Botrytis cinerea* PMFKG-F33 and 4 yeast species *Candida albicans* ATCC 10231, *Candida albicans* (clinical isolate); *Rhodotorula sp.* PMFKG-F27 and *Saccharomyces boulardii* PMFKG-P34. All clinical isolates were a generous gift from the Institute of Public Health, Kragujevac. The other microorganisms were provided from the collection of the Laboratory of Microbiology, Faculty of Science, University of Kragujevac.

#### 3.5.3. Suspension Preparation

The bacterial suspensions and the yeast suspension were prepared by the direct colony method [52]. The colonies were taken directly from the plate and were suspended in 5 mL of sterile 0.85% saline. The turbidity of the initial suspension was adjusted by comparing with 0.5 McFarland’s standard (0.5 mL 1.17% w/v BaCl_2_ × 2H_2_O + 99.5 mL 1% w/v H_2_SO_4_). When adjusted to the turbidity of the 0.5 McFarland’s standard, the bacteria suspension contains about 10^8^ colony forming unites (CFU)/mL while the suspension of yeast contains 10^6^ CFU/mL. 1:100 dilutions of the initial suspension were additionally prepared into sterile 0.85% saline. The suspensions of fungal spores were prepared by a gentle stripping of the spore from the slopes with growing aspergilli. The resulting suspensions were 1:1000 diluted in sterile 0.85% saline.

#### 3.5.4. Microdilution Method

The antimicrobial activity was tested by determining the minimum inhibitory concentration (MIC) and minimum microbicidal concentration (MMC) using a microdilution method with resazurin [42]. The 96-well plates were prepared by dispensing 100 μL of nutrient broth, Mueller–Hinton broth for bacteria and Sabouraud dextrose broth for fungi and yeasts, into each well. A 100 μL from the stock solution of the tested compound (concentration of 80 mg/mL) was added into the first row of the plate. Then, twofold, serial dilutions were performed by using a multichannel pipette. The obtained concentration range was from 40 to 0.0098 mg/mL. A 10 μL of the diluted bacterial, yeast suspension and suspension of spores was added to each well to give a final concentration of 5 × 10^5^ CFU/mL for bacteria and 5 × 10^3^ CFU/mL for fungi and yeast. Finally, a 10 μL resazurin solution was added to each well inoculated with bacteria and yeast. Resazurin is an oxidation–reduction indicator used for the evaluation of microbial growth. It is a blue non-fluorescent dye that becomes pink and fluorescent when reduced to resorufin by oxidoreductases within viable cells. The inoculated plates were incubated at 37 °C for 24 h for bacteria, 28 °C for 48 h for the yeast and 28 °C for 72 h for filamentous fungi. MIC was defined as the lowest concentration of tested substance that prevented resazurin color change from blue to pink. For filamentous fungi, MIC values of the tested substance were determined as the lowest concentration that visibly inhibited mycelia growth.

Doxycycline and fluconazole were used as a positive control. A solvent control test was performed to study an effect of 5% DMSO on the growth of a microorganism. It was observed that 5% DMSO did not inhibit the growth of a microorganism. Also, in the experiment, the concentration of DMSO was additionally decreased because of the twofold serial dilution assay (the working concentration was 2.5% and lower). Each test included growth control and sterility control. All tests were performed in duplicate and MICs were constant.

The minimum bactericidal and fungicidal concentration was determined by plating 10 μL of samples from the wells, where no indicator color change was recorded, on the nutrient agar medium. At the end of the incubation period the lowest concentration with no growth (no colony) was defined as minimum microbicidal concentration.

### 3.6. In Vitro Antiproliferative Assays

#### 3.6.1. Cell Lines

The colon cancer adenocarcinoma cell line HCT-116 was obtained from the American Tissue Culture Collection (Manassas, VA, USA). These cells were maintained in Dulbecco’s Modified Eagle Medium (DMEM) (Gibco, Invitrogen, New York, USA) containing 10% fetal bovine serum (FBS), 100 IU/mL penicillin and 100 μg/mL streptomycin. The cells were grown in 75 cm^2^ flasks (SARSTEDT AG & Co., Nümbrecht, Germany) and after a few passages the cells were seeded in 96-well plate. Cells were cultured in a humidified atmosphere of 5% CO_2_ at 37 °C. The cell numbers were determined by trypan blue exclusion.

#### 3.6.2. MTT Assay

After 24 and 72 h of treatment, the cell viability was determined by the MTT (3-[4,5-dimethylthiazol-2-yl]-2,5-diphenyltetrazolium bromide) reduction assay [45]. MTT assay is a test of cell proliferation based on colored reaction of mitochondrial dehydrogenase from living cells with MTT. HCT-116 cells were seeded in a 96-well plate (10^4^ cells per well) and cultivated for 24 h. After that the cells were treated with 100 μL of diluted lichen extracts (concentration ranged from 50 to 1000 μg/mL) 24 and 72 h. The untreated cells served as a control. At the end of the treatment period, MTT (final concentration 5 mg/mL in PBS) (Sigma, St. Louis, USA) was added to each well, which was then incubated at 37 °C in 5% CO_2_ for 2 h. The colored crystals of the produced formazan were dissolved in DMSO (dimethyl sulfoxide) (Sigma, St. Louis, USA). The absorbance was measured at 550 nm on Microplate Reader. Cell proliferation (% viability cells) was calculated as a ratio of the absorbance of the treated group divided by the absorbance of the control group, multiplied by 100 to give percentage of the proliferation.

The antiproliferative effect of each extract was expressed by IC_50_(inhibitory dose which inhibits 50% of cell growth) and by the magnitude of the maximal effect in exposed cells. The IC_50_values were calculated from calibration curve by a CalcuSyn computer program.

#### 3.6.3. Fluorescence Microscopic Analysis of Cell Death (AO/EB) Double Staining

For the analysis of cell death, we used fluorescent assays of the acridine orange/ethidium bromide (AO/EB) double staining. Acridine orange is taken up by both viable and nonviable cells which emit green fluorescence if intercalated into double stranded nucleic acid (DNA) or red fluorescence if bound to single stranded nucleic acid (RNA). Ethidium bromide is taken up only by nonviable cells which emit red fluorescence by intercalation into DNA [46].

HCT-116 cells were grown in a 6-well plate (3 × 10^4^ cells per well) for 24 h. After that, 2 mL (250 μg/mL) of each lichen’s methanol extracts were added and the cells were cultivated for 24 and 72 h. The untreated cells served as a control. The incubation was performed at 37 °C in an atmosphere of 5% CO_2_ and 95% of relative humidity. After 24 and 72 h of treatment, 200 μL of dye mixture (100 μL of 100 mg/mL AO and 100 μL of 100 mg/mL EB in distilled water) was added to each well. The suspension was immediately (fast uptake) examined by fluorescence microscopy (NICON Eclipse Ti) at 400× magnification. A minimum of 300 cells was counted in every sample.

#### 3.6.4. Statistical Analysis

The data were expressed as the means ± standard deviation (SD). All statistical analyses were performed using SPSS package (SPSS for Windows, ver. 17, 2008) (Chicago, IL, USA). Mean differences were established by Student’s *t*-test. Data were analyzed using one-way analysis of variance (ANOVA). In all cases p values <0.05 were considered statistically significant.

## 4. Conclusion

The antioxidant, antimicrobial and antiproliferative activities of the five lichen species from Familia *Parmeliacea* **(***Parmelia sulcata*, *Flavoparmelia caperata*, *Evernia prunastri*, *Hypogymnia physodes* and *Cladonia foliacea*) were demonstrated. The best results were obtained from *Hypogymnia physodes* and *Cladonia foliacea.* Further work will be done on the isolation and purification of active components in these species.

## Figures and Tables

**Table 1.** The comparison of the total phenolic content, the total flavonoid content and the antioxidant activity of the lichen extracts.

**Lichen species**	Total phenolic content ^1, *^	Total flavonoid content ^2, *^	Antioxidant Activity ^3, *^	**Chemical composition**
*Parmelia sulcata*	88.25 ± 1.02	44.43 ± 1.22	584.22 ± 1.28	Arabinitol, atraric acid, atranol, α-tocopherol, β-sitosterol, ergosterol, oleic acid, linolenic acid, nonacosane, linoleic acid, palmitic acid, methyl haematommate, olivetol, lichesterol, stearic acid, salazinic acid, divaricatic acid [27,28]
*Flavoparmelia caperata*	90.83 ± 0.98	33.55 ± 0.93	549.01 ± 1.69	Usnic acid, atraric acid, arabinitol, atranol, orcinol, lichesterol, ergosterol, protocetraric acid, caperatic acid [21,27,29]
*Evernia prunastri*	80.73 ± 1.25	27.46 ± 0.78	>1000.00	Atraric acid, orcinol, usnic acid, methyl orsellinate, orcinol monomethylether, methyl haematommate, atranol, arabinitol, sparassol, orsellinic acid, linoleic acid, oleic acid, stearic acid, palmitic acid, lichesterol, ergosterol, evernic acid [28,30]
*Hypogymnia physodes*	141.59 ± 1.12	20.14 ± 0.81	45.57 ± 1.35	Olivetol, atraric acid, olivetonide, olivetonic acid, atranol, ergosterol, methyl haematommate, lichesterol, oleic acid, stearic acid, palmitic acid, linoleic acid, orcinol, α-tocopherol, hloroatranol, physodic acids, physodalic acid, isophysodic acid, 3-hydroxyphysodic acid, 2′-O-methylphysodic acid [27,28,30,31]
*Cladonia foliacea*	78.12 ± 1.31	28.22 ± 0.59	>1000.00	Usnic acid, atranorin, fumarprotocetraric acid [32]

1 Total phenolic content expressed as gallic acid equivalent (mg GA/g of extract);

2 Total flavonoid content expressed as rutin equivalent (mg Ru/g of extract);

3 Antioxidant activity expressed as IC_50_values of DPPH scavenging activity of lichen extracts (μg/mL);

* Each value in the table was obtained by calculating the average of three analysis ± standard deviation.

**Table 2.** The antibacterial activity of the methanol extracts of lichens *Parmelia sulcata*, *Flavoparmelia caperata* and *Evernia prunastri.*

**Species**	***Parmelia sulcata***		***Flavoparmelia caperata***		***Evernia prunastri***		**Doxycycline**	
	MIC *	MMC *	MIC *	MMC *	MIC *	MMC *	MIC *	MMC *
*Sarcina lutea*	3.13 × 10^−1^	3.13 × 10^−1^	7.81 × 10^−2^	7.81 × 10^−2^	7.81 × 10^−2^	7.81 × 10^−2^	<4.48 × 10^−4^	3.75 ×10^−3^
*Enterococcus faecalis*	10.00	10.00	10.00	10.00	10.00	10.00	7.81 × 10^−3^	6.25 × 10^−2^
*Enterococcus faecalis* ATCC 29212	5.00	5.00	7.81 × 10^−2^	1.56 × 10^−1^	3.13 × 10^−1^	3.13 × 10^−1^	7.81 × 10^−3^	6.25 × 10^−2^
*Bacillus subtilis*	1.56 × 10^−1^	3.13 × 10^−1^	1.95 × 10^−2^	3.91 × 10^−2^	3.91 × 10^−2^	3.91 × 10^−2^	1.12 × 10^−4^	1.95 × 10^−3^
*Bacillus subtilis* ATCC 6633	7.81 × 10^−2^	1.56 × 10^−1^	1.95 × 10^−2^	1.95 × 10^−2^	3.91 × 10^−2^	7.81 × 10^−2^	1.95 × 10^−3^	3.13 × 10^−2^
*Bacillus cereus*	3.13 × 10^−1^	3.13 × 10^−1^	3.91 × 10^−2^	3.91 × 10^−2^	7.81 × 10^−2^	7.81 × 10^−2^	9.77 × 10^−4^	7.81 × 10^−3^
*Staphylococcus aureus*	3.13 × 10^−1^	1.25	1.56 × 10^−1^	1.56 × 10^−1^	1.56 × 10^−1^	3.13 × 10^−1^	4.48 × 10^−4^	7.81 × 10^−3^
*Staphylococcus aureus* ATCC 25923	10.00	10.00	1.56 × 10^−1^	3.13 × 10^−1^	1.56 × 10^−1^	6.25 × 10^−1^	2.24 × 10^−4^	3.75 × 10^−3^
*Escherichia coli*	5.00	5.00	10.00	10.00	10.00	10.00	7.81 × 10^−3^	1.56 × 10^−2^
*Escherichia coli* ATCC 25922	5.00	5.00	10.00	10.00	10.00	10.00	1.56 × 10^−2^	3.13 × 10^−2^
*Pseudomonas aeruginosa*	2.50	5.00	2.50	10.00	2.50	10.00	2.50 × 10^−1^	2.50 × 10^−1^
*Pseudomonas aeruginosa* ATCC 27853	6.25 × 10^−1^	5.00	6.25 × 10^−1^	10.00	2.50	20.00	6.25 × 10^−2^	1.25 × 10^−1^
*Proteus mirabilis*	5.00	5.00	2.50	5.00	5.00	5.00	5.00	2.50 × 10^−1^
*Salmonella enterica*	5.00	5.00	10.00	20.00	10.00	20.00	1.56 × 10^−2^	3.13 × 10^−2^
*Salmonella typhymirium*	5.00	5.00	10.00	10.00	10.00	20.00	1.56 × 10^−2^	1.25 × 10^−1^

* Minimum inhibitory concentration (MIC) and minimum microbicidal concentration (MMC) values for lichen extracts and antibiotic are given as mg/mL. Antibiotic: Doxycycline.

**Table 3.** The antibacterial activity of the methanol extracts of lichens *Hypogymnia physodes* and *Cladonia foliacea.*

**Species**	***Hypogymnia physodes***		***Cladonia foliacea***		**Doxycycline**	
	MIC *	MMC *	MIC *	MMC *	MIC *	MMC *
*Sarcina lutea*	<9.80 × 10^−3^	<9.80 × 10^−3^	1.95 × 10^−2^	3.91 × 10^−2^	<4.48 × 10^−4^	3.75 × 10^−3^
*Enterococcus faecalis*	5.00	10.00	20.00	20.00	7.81 × 10^−3^	6.25 × 10^−2^
*Enterococcus faecalis* ATCC 29212	7.81 × 10^−2^	7.81 × 10^−2^	3.91 × 10^−2^	3.91 × 10^−2^	7.81 × 10^−3^	6.25 × 10^−2^
*Bacillus subtilis*	7.81 × 10^−2^	7.81 × 10^−2^	<9.80 × 10^−3^	<9.80 × 10^−3^	1.12 × 10^−4^	1.95 × 10^−3^
*Bacillus subtilis* ATCC 6633	3.91 × 10^−2^	3.91 × 10^−2^	<9.80 × 10^−3^	<9.80 × 10^−3^	1.95 × 10^−3^	3.13 × 10^−2^
*Bacillus cereus*	7.81 × 10^−2^	7.81 × 10^−2^	<9.8 × 10^−3^	<9.80 × 10^−3^	9.77 × 10^−4^	7.81 × 10^−3^
*Staphylococcus aureus*	3.91 × 10^−2^	7.81 × 10^−2^	3.91 × 10^−2^	7.81 × 10^−2^	4.48 × 10^−4^	7.81 × 10^−3^
*Staphylococcus aureus* ATCC 25923	3.13 × 10^−1^	6.25 × 10^−1^	7.81 × 10^−2^	7.81 × 10^−2^	2.24 × 10^−4^	3.75 × 10^−3^
*Escherichia coli*	2.50	2.50	10.00	20.00	7.81 × 10^−3^	1.56 × 10^−2^
*Escherichia coli* ATCC 25922	5.00	5.00	10.00	20.00	1.56 × 10^−2^	3.13 × 10^−2^
*Pseudomonas aeruginosa*	6.25 × 10^−1^	1.25	5.00	20.00	2.50 × 10^−1^	>2.50 × 10^−1^
*Pseudomonas aeruginosa* ATCC 27853	6.25 × 10^−1^	2.50	1.25	2.50	6.25 × 10^−2^	1.25 × 10^−1^
*Proteus mirabilis*	6.25 × 10^−1^	6.25 × 10^−1^	5.00	10.00	2.50 × 10^−1^	>2.50 × 10^−1^
*Salmonella enterica*	1.25	10.00	10.00	20.00	1.56 × 10^−2^	3.13 × 10^−2^
*Salmonella typhymirium*	1.25	2.50	10.00	20.00	1.56 × 10^−2^	1.25 × 10^−1^

* Minimum inhibitory concentration (MIC) and minimum microbicidal concentration (MMC) values for lichen extracts and antibiotic are given as mg/mL. Antibiotic: Doxycycline.

**Table 4.** The antifungal activity of the methanol extracts of lichens *Parmelia sulcata, Flavoparmelia caperata*, *and Evernia prunastri*.

**Species**	***Parmelia sulcata***		***Flavoparmelia caperata***		***Evernia prunastri***		**Fluconazol**	
	MIC *	MMC *	MIC *	MMC *	MIC *	MMC *	MIC *	MMC *
*Candida albicans*	2.50	20.00	2.50	10.00	2.50	10.00	6.25 × 10−^2^	1.00
*Candida albicans* ATCC 10231	5.00	20.00	10.00	20.00	2.50	10.00	3.13 × 10−^2^	1.00
*Rhodotorula sp.*	2.50	2.50	2.50	5.00	1.25	2.50	6.25 × 10−^2^	1.00
*Saccharomyces boulardii*	10.00	20.00	10.00	40.00	5.00	10.00	3.13 × 10−^2^	1.00
*Penicillium italicum*	2.50	5.00	2.50	5.00	1.25	5.00	1.00	1.00
*Penicillium chrysogenum*	2.50	2.50	6.25 × 10−^1^	2.50	1.25	1.25	6.25 × 10−^2^	5.00 × 10−^1^
*Penicillium digitatum*	1.25	1.25	5.00	10.00	1.25	1.25	3.13 × 10−^2^	3.13 × 10−^2^
*Botrytis cinerea*	10.00	20.00	40.00	40.00	1.25	5.00	3.13 × 10−^2^	5.00 × 10−^1^
*Trichothecium roseum*	6.25 × 10−^1^	6.25 × 10−^1^	2.50	5.00	1.25	5.00	5.00 × 10−^1^	5.00 × 10−^1^
*Aspergillus niger*	10.00	20.00	2.50	20.00	1.25	5.00	5.00 × 10−^1^	1.00
*Aspergillus niger* ATCC 16404	2.50	2.50	10.00	40.00	1.56 × 10−^1^	1.56 × 10−^1^	6.25 × 10−^2^	6.25 × 10−^2^
*Aspergillus restrictus*	6.25 × 10−^1^	6.25 × 10−^1^	5.00	5.00	1.25	1.25	5.00 × 10−^1^	2.00
*Aspergillus fumigatus*	<9.80 × 10−^3^	<9.80 × 10−^3^	1.25	1.25	1.25	1.25	5.00 × 10−^1^	1.00
*Aspergillus flavus*	1.25	2.50	2.50	5.00	1.25	5.00	1.00	1.00

* Minimum inhibitory concentration (MIC) and minimum microbicidal concentration (MMC) values for lichen extracts and antimycotic are given as mg/mL. Antimycotic: Fluconazole.

**Table 5.** The antifungal activity of the methanol extracts of lichens *Hypogymnia physodes* and *Cladonia foliacea.*

**Species**	***Hypogymnia physodes***		***Cladonia foliacea***		**Fluconazol**	
	MIC *	MMC *	MIC *	MMC *	MIC *	MMC *
*Candida albicans*	5.00	20.00	5.00	20.00	6.25 × 10^−2^	1.00
*Candida albicans* ATCC 10231	5.00	10.00	5.00	20.00	3.13 × 10^−2^	1.00
*Rhodotorula sp.*	1.25	2.50	1.25	5.00	6.25 × 10^−2^	1.00
*Saccharomyces boulardii*	10.00	20.00	5.00	40.00	3.13 × 10^−2^	1.00
*Penicillium italicum*	1.25	2.50	2.50	10.00	1.00	1.00
*Penicillium chrysogenum*	6.25 × 10^−1^	2.50	1.25	5.00	6.25 × 10^−2^	5.00 × 10^−1^
*Penicillium digitatum*	1.25	1.25	5.00	5.00	3.13 × 10^−2^	3.13 × 10^−2^
*Botrytis cinerea*	10.00	10.00	5.00	20.00	3.13 × 10^−2^	5.00 × 10^−1^
*Trichothecium roseum*	1.25	2.50	10.00	20.00	5.00 × 10^−1^	5.00 × 10^−1^
*Aspergillus niger*	5.00	10.00	20.00	20.00	5.00 × 10^−1^	1.00
*Aspergillus niger* ATCC 16404	<9.80 × 10^−3^	<9.80 × 10^−3^	10.00	10.00	6.25 × 10^−2^	6.25 × 10^−2^
*Aspergillus restrictus*	1.25	1.25	2.50	10.00	5.00 × 10^−1^	2.00
*Aspergillus fumigatus*	1.25	1.25	3.13 × 10^−1^	2.50	5.00 × 10^−1^	1.00
*Aspergillus flavus*	1.25	1.25	2.50	10.00	1.00	1.00

* Minimum inhibitory concentration (MIC) and minimum microbicidal concentration (MMC) values for lichen extracts and antimycotic are given as mg/mL. Antimycotic: Fluconazole.

**Table 6.** The growth inhibitory effects of the methanol extracts on HCT-116 cells expressed as IC_50_values (μg/mL).

**Lichen extract**	**IC_50_ (μg/mL)**	
	**24 h**	**72 h**
*Parmelia sulcata*	608.83 ± 36.52	913.03 ± 63.91
*Flavoparmelia caperata*	397.64 ± 19.88	229.55 ± 13.77
*Evernia prunastri*	303.47 ± 15.25	295.64 ± 23.65
*Hypogymnia physodes*	253.72 ± 17.76	102.40 ± 7.16
*Cladonia foliacea*	265.55 ± 13.27	122.47 ± 9.79

**Table 7.** Apoptosis of HCT-116 cells induced by 24 h exposure to the lichen extracts.

**Lichen extract**	**Viable cells (%)**	**Early apoptotic cells (%)**	**Late apoptotic cells (%)**	**Necrotic cells (%)**
*None*	96.80	3.20	-	-
*Parmelia sulcata*	65.36	34.02	0.41	0.20
*Flavoparmelia caperata*	68.08	31.91	-	-
*Evernia prunasti*	66.48	33.51	-	-
*Hypogymnia physodes*	28.88	42.22	11.11	17.78
*Cladonia foliacea*	48.99	49.66	-	1.34

**Table 8.** Apoptosis of HCT-116 cells induced by 72 h exposure to the lichen extracts.

**Lichen extract**	**Viable cells (%)**	**Early apoptotic cells (%)**	**Late apoptotic cells (%)**	**Necrotic cells (%)**
*None*	71.12	28.88	-	-
*Parmelia sulcata*	73.66	26.20	0.20	-
*Flavoparmelia caperata*	1.80	59.72	38.46	-
*Hypogimnia physodes*	-	14.21	53.15	32.62
*Evernia prunasti*	65.60	34.39	-	-
*Cladonia foliacea*	-	51.59	48.40	-
